# Genealogy of Conjugated Acyclic Polyenes

**DOI:** 10.3390/molecules22060896

**Published:** 2017-05-29

**Authors:** Haruo Hosoya

**Affiliations:** Department of Information Sciences, Faculty of Science, Ochanomizu University (Emeritus) Otsuka 2-1-1, Bunkyo-ku, Tokyo 112-8610, Japan; hosoya.haruo@ocha.ac.jp

**Keywords:** total π-electronic energy of HMO, conjugated polyene, topological index, Hosoya index, structure-stability relation, Kekulé structure, mean length of conjugation, cross-conjugation, branching

## Abstract

Based on the total π-electron energies *E*_π_s of Hückel Molecular Orbital (HMO) method for all the possible isomers of conjugated acyclic polyenes (C_2*n*_H_2*n*+2_) up to *n* = 7, the structure–stability relation of the possible isomers was analyzed. It was shown that the mean length of conjugation *L* can roughly predict the ordering of stability among isomers, while the *Z*-index, or Hosoya-index, can almost perfectly reproduce their stability. Further, the genealogy of the conjugated acyclic polyene family was obtained by drawing systematic diagrams connecting these isomers of different *n*, and governed by several simple rules. Namely, the stability change of a given isomer in the genealogy connecting *n* and *n* + 1 polyenes can be classified into three different modes of vinyl addition (elongation, inner and outer branching) and horn growing, i.e., substitution of –HC=CH– moiety with –HC(=CH_2_)–C(=CH_2_)H–. By using the *Z*-index, we can extend this type of discussion to polyene radicals and even to “cross-conjugated” cyclic polyenes containing only one odd-membered cycle, such as radialene and fulvene.

## 1. Introduction

The successful isolation and identification of the tremendously large family of organic compounds, even limited to hydrocarbons, seems to have established the logical structure of the kingdom of organic chemistry, which is opening its open gates to other fields of science spanning from biology to astronomy, information technology, and general physics. However, due to the scarcity of the isolated conjugated acyclic polyene molecules, even for the smallest members, the present status of organic chemists’ understanding of the structure–activity relationship and mathematics underlying the whole family of conjugated acyclic polyenes is rather low. Unfortunately, without paying attention to the essence of quantum theory, they are still playing with the old-style resonance theory originally proposed by the chemists in “pre-quantum chemistry age”.

In order to steer towards the right direction, the present author has published several papers aimed at understanding and justifying the organic electron theory, mainly involving conjugated polyenes by using the graph-theoretical molecular orbital (GTMO) method. The classical concept of cross-conjugation, if properly appreciated with a slight modification, will play an important role for understanding the correct part of the conventional organic electron theory [1,2,3,4,5,6].

Fortunately, however, novel methods for synthesizing dendralenes (vide infra) and the related hydrocarbons have recently been discovered and several researchers have reconsidered the importance of the role of cross-conjugation in organic chemistry [7,8,9,10,11,12].

In the present paper, the genealogy and mathematical structure of the whole family of conjugated acyclic polyene molecules are explained in various levels of logic from high school chemistry (without wavefunction) to sophisticated mathematical chemistry (with perturbation theory) only by using the Kekulé structure, total π-electron energy of the HMO method, and topological index *Z* (the so-called Hosoya-index) [13,14].

## 2. Preliminary Discussions

### 2.1. Planar Conjugated Acyclic Polyene Isomers

We are concerned only with planar conjugated acyclic polyenes. First, consider the series of linear polyenes, i.e., ethylene **1**, butadiene **2**, linear hexatriene **3-1**, etc., which are growing mostly in zigzag form, or all-trans conformation up to polyacetylene. The longer the chain length, the more their π-electronic stability increases. Above hexatriene, we need to consider isomers, such as 3-methylene-1,4-pentadiene, or 3-dendralene **3-2**, which is known also as the smallest entity of cross-conjugated hydrocarbon [15]. According to the analysis of electron diffraction, **3-2** takes a slightly distorted ct (*cis*-*trans*) conformation [16]—in accordance with the results of molecular mechanics and ab initio calculation [17].


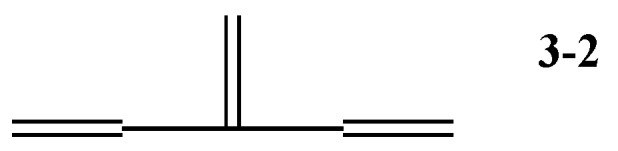


However, one can easily conjecture this fact just by estimating the repulsion between non-bonded hydrogen atoms. Thus, one can develop our naïve discussion on the relative stabilities of “almost planar” conjugated acyclic polyene isomers up to, say tetradecaheptaene, whose most crowded isomer might be among its 96 isomers. Here and from now on, we will not consider their complicated *cis*–*trans* conformations, helical structures, and sophisticated isomer counting involving radicals and triple bonds [18,19,20].


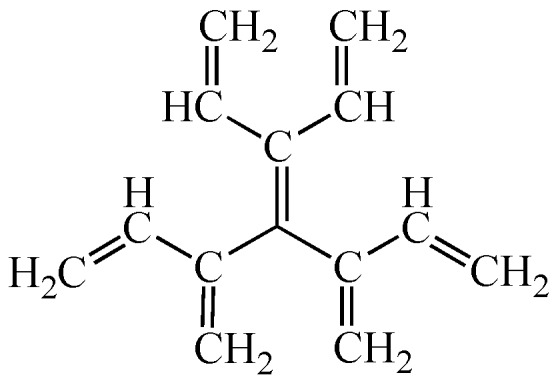


It may be a worthwhile exercise for students to obtain the possible isomer numbers for smaller conjugated acyclic polyenes of “naïve” sp^2^-type as given in Table 1 [21], because it is difficult to check these important numbers in any of the available standard textbooks of organic chemistry past and present. Although it would be impossible to experimentally ascertain these numbers, we need to know both the topographical and mathematical structure of this half hypothetical, but half realistic kingdom of conjugated acyclic polyenes, the genealogy of which we are going to clarify in this paper.

Recall that in physical organic chemistry, we have established the grand conceptual kingdom of aromatic and anti-aromatic hydrocarbons by using not only the experimentally obtained results but also assuming hypothetical aromatic and anti-aromatic compounds. The present author considers the logical base of this kingdom to still be shaky in this modern age, because logically and mathematically it should be constructed on the firm basis of the world of conjugated acyclic polyenes. The only common understanding among a majority of chemists is that the linear polyene is the most stable and dendralene is the least stable among the isomers.

Although, on this structure–stability relation of conjugated acyclic polyenes, Gutman has shown that the isomer of the following type is the most stable among the branched isomers [22], this fact does not yet seem to be widely known to organic chemists.


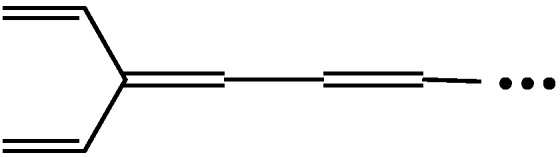


On the other hand, Gineityte’s discussion is too sophisticated and specific to be followed by a majority of chemists [11].

### 2.2. Mean Length of Conjugation

Now consider the relative stability of two isomers of hexatrienes. Due to the short conjugated path of the π-electronic system, 3-dendralene **3-2** is less stable than linear **3-1** [15]. What about the relative stability of four isomers of octatetraene, **4-1**~**4-4**?

Gutman’s assertion can be supported by the *E*_π_ calculation [4,6] as in the following order.


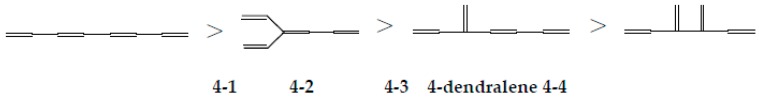


Except for the order within the middle two isomers, the number of tertiary carbon atoms, *T*, can predict the relative order of their stability. Gutman et al. also pointed out the important role of *T* in discussing the relative stability of conjugated acyclic polyenes [23]. Although the counting of Dewar structures (with a long bond connecting a pair of disjointed carbon atoms) can predict the correct order of stability [10], unfortunately this method is not explained in any elementary chemistry textbooks.

**Figure 1:**
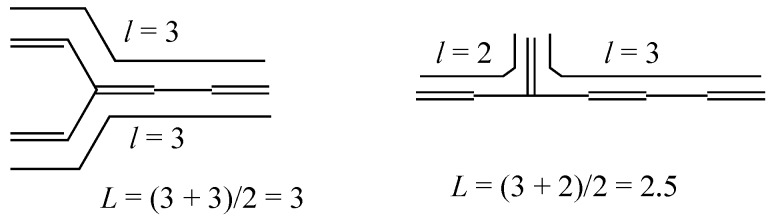
Calculation of mean length of conjugation *L* for **4-2** and **4-3**, both with *T* = 1.

The present author has shown the effectiveness of the “mean length of conjugation”, *L* [4,6], for predicting the relative stability between **4-2** and **4-3**, which is easily enumerated from the unique Kekulé structure as exemplified in Figure 1. Here, each *l* is the largest number of C=C bonds in a linearly conjugated acyclic polyene moiety that consists of alternating *l* C=C and *l*–1 C–C bonds.

### 2.3. Hückel Molecular Orbital Method and E_π_

In order to settle the problem of relative stability among conjugated polyene isomers, let us turn to the HMO method, which is the easiest and most reliable technique at hand. The total π-electron energy, *E*_π_, is defined as the double of the sum of the occupied orbital energies, {*x_n_*}s as in

(1) $$E_{\pi }=2\sum_{n=1}^{\mathrm{occ}}x_{n}$$

The {*x_n_*}s are obtained as the solution of the characteristic polynomial, P_G_(*x*) = 0, for

(2) $$P_{G}\left(x\right)={\left(-1\right)}^{N}\det(A-xE)$$

of molecular graph G, representing the topology of the carbon atom skeleton of the polyene molecule, where **A** and **E** are respectively the adjacency matrix of G and unit matrix of the order *N*, the number of carbon atoms of G, and det means to take the determinant of the matrix given in the parentheses. The adjacency matrix **A** is defined to be for G.

(3) $$A_{ij}=\{\begin{array}{cc} 1: & i\;\mathrm{and}\;j\;\text{are neighbors}\\ 0: & \mathrm{otherwise} \end{array}$$

In Table 2, *E*_π_ and P_G_(*x*) of the four isomers of octatetraene are compared with *T* and *L* values. Needless to say, regarding *T*, the correlation of *L* with *E*_π_ is rather good. However, the quantity *Z*, which will be explained below, is found to be perfectly correlated with *E*_π_. The correlation coefficient ρ is almost unity for *Z*, while ρ is 0.981 for *L*.

### 2.4. Topological Index

In 1971, the present author proposed to define the topological index *Z* [13,24], which is now generally called the Hosoya-index [25,26,27,28], for graph G (See Table 2 for the molecular graphs of octatetraenes) as the sum of the non-adjacent number, *p*(G,*k*), where the number of ways for choosing *k* non-adjacent edges from G as

(4) $$Z=\sum_{k=0}^{\left[N/2\right]}p(G,k)$$

and also found that for tree graphs (representing acyclic molecules), P_G_(*x*) can be expressed just by using the *p*(G,*k*) numbers as

(5) $$P_{G}\left(x\right)=\sum_{k=0}^{\left[N/2\right]}{\left(-1\right)}^{k}p(G,k)x^{N-2k}\;\left(G\in \mathrm{tree}\right)$$

Now, as seen in Table 2, all the *Z* values are equal to the sum of the absolute values of the coefficients of P_G_(*x*).

Originally, *p*(G,*k*) and *Z* were proposed for analyzing the thermodynamic properties of the structural isomers of saturated hydrocarbons. However, as inferred from the close relation with HMO as (5), *Z* was found to be well correlated with *E*_π_ αs

*E*_π_ ∝ log *Z* (6)

which was proven by using the perturbation theory of Longuet-Higgins [14,29]. When the relative stability among isomeric hydrocarbons is discussed, we are allowed to use a simpler relation [2,4,6],

*E*_π_ = *a Z* + *b*. (7)

A convenient method for calculating the *Z* value of branched tree graphs will be explained in Appendix A.

In the same year as the debut of the *Z*-index, Hess and Schaad proposed an empirical recipe for reproducing *E*_π_ for conjugated acyclic polyenes by adding the contribution of eight types of bonds, such as H_2_C=CH, HC=CH, etc., which gave tremendously good results as shown by the *E*(H&S) values in Table 2 [30]. Their recipe gives rather reasonable results even for conjugated cyclic hydrocarbons to estimate their aromatic character [31,32,33]. However, it is very difficult to draw any physico-chemical meaning of each parameter, and further, for larger hydrocarbons with *N* ≥ 10 the discriminative power suddenly drops down, as will be shown later.

By scrutinizing Table 2, it can be inferred that the structure–stability relation for the conjugated acyclic polyenes is governed by rather simple and straightforward rules. This optimistic inference is further strengthened by checking the data for larger conjugated polyenes [4,6].

**Figure 2:**
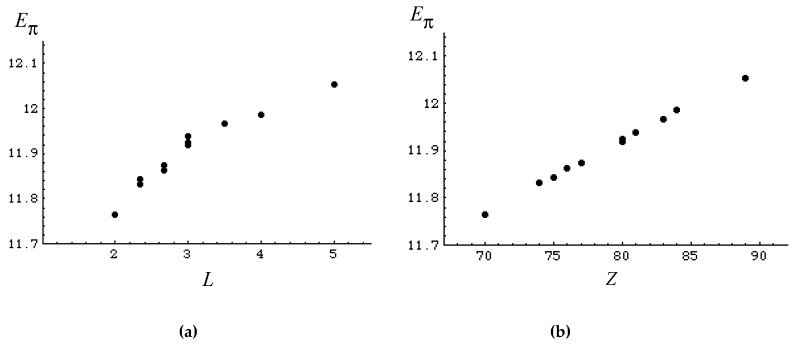
Correlation of *E*_π_’s of eleven isomers of decapentaenes with *L* (**a**) and *Z* (**b**).

Namely, for example, Figure 2a,b show the correlation of *E*_π_’s of eleven isomers of decapentaenes, C_10_H_12_, with *L* and *Z*. Their ρ values are, respectively, as high as 0.954 and 0.999.

**Figure 3:**
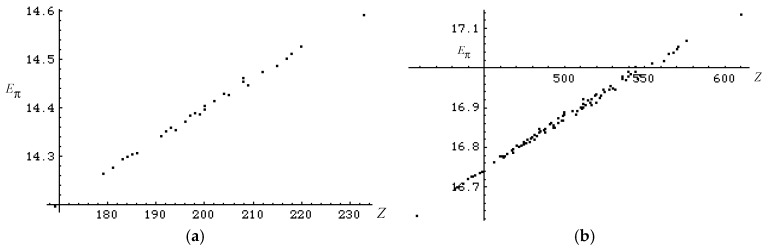
Correlation between *Z* and *E*_π_ for isomers of (**a**) 30 dodecahexaenes and (**b**) 96 tetradecaheptaenes.

Further, for much larger conjugated acyclic polyenes with *n* = 12 and 14, the good correlation between *Z* and *E*_π_ does not change as shown in Figure 3a,b, where, respectively, one and three isospectral pairs are found. Now we are going to analyze the genealogy of conjugated acyclic polyenes.

## 3. Results and Discussion

### 3.1. Vinyl Addition and Horn Growing

**Figure 4:**
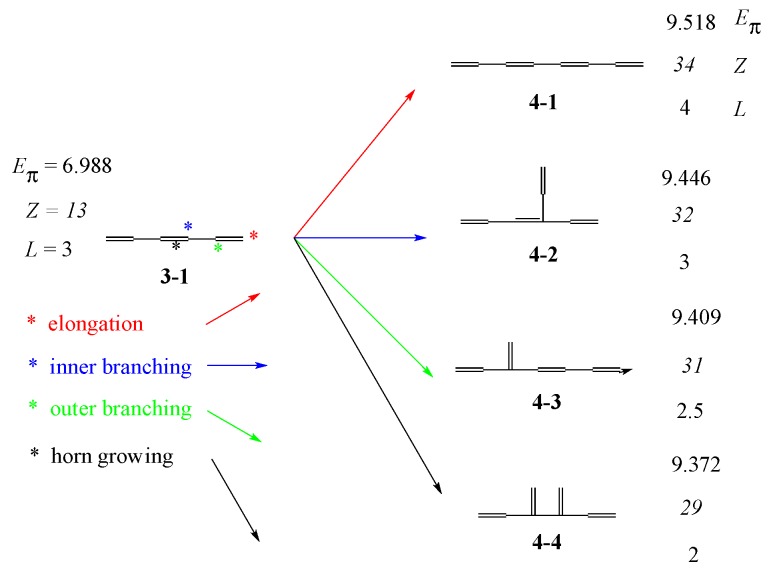
Three kinds of addition of a vinyl group and “horn growing” to **3-1** derive all the four isomers of C_8_H_10_.

With these results in mind, reconsider the relation between linear hexatriene **3-1** and four octatetranes, three of which can be derived by adding a vinyl group, CH_2_=CH–, to **3-1**. As seen in Figure 4, the three isomers, **4-1**, **4-2**, and **4-3,** can be derived, respectively, by the addition at the positions 1 (red), 3 (blue), and 2 (green), counted from the terminal carbon atom. Let us call these step-up growing processes, elongation, inner branching, and outer branching, respectively.

**Figure 5:**
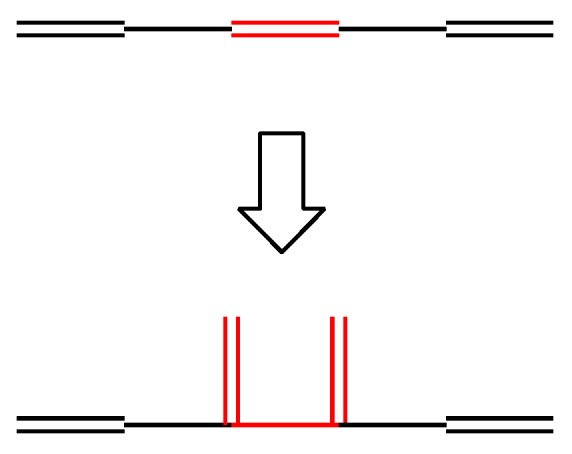
Horn growing.

The least stable **4-4** cannot be derived from **3-1** by the addition of a vinyl group, but can be derived from **3-2** by outer branching. Similarly, one can derive all the isomers of a given *N* from the set of *N*–2 isomers by using the elongation and two types of branching. However, for understanding the whole genealogy and the structure–stability relation hidden there, let us consider a slightly different growing type, such as the one shown in Figure 5, which has already been given in Figure 4 and may be called “horn growing”.

**Figure 6:**
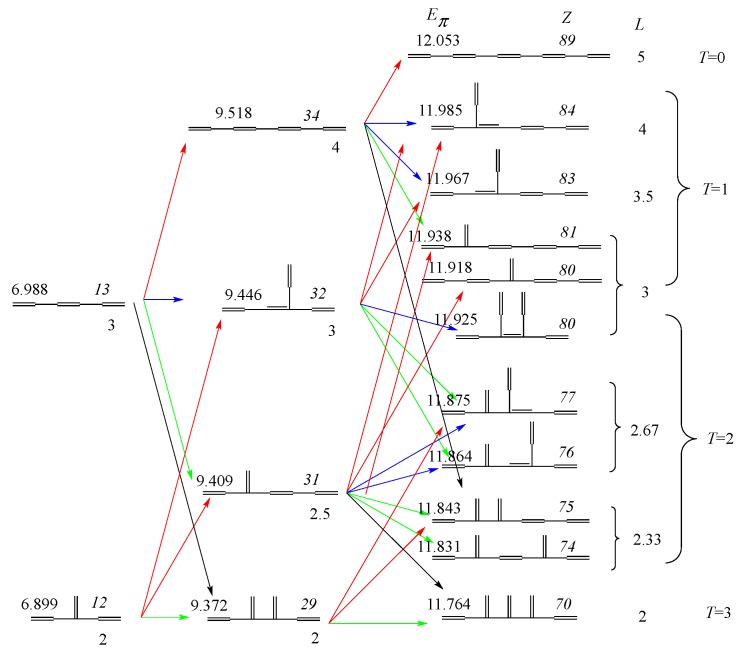
Genealogy of hexatrienes~decapentaene. Meaning of colors is the same as in Figure 4.

Now, we can draw the whole genealogy of three generations of conjugated acyclic polyenes, C_6_H_8_~C_10_H_12_, as in Figure 6, where *E*_π_ values, *Z*-index (in italics), *L*, and *T* are given.

**Figure 7:**
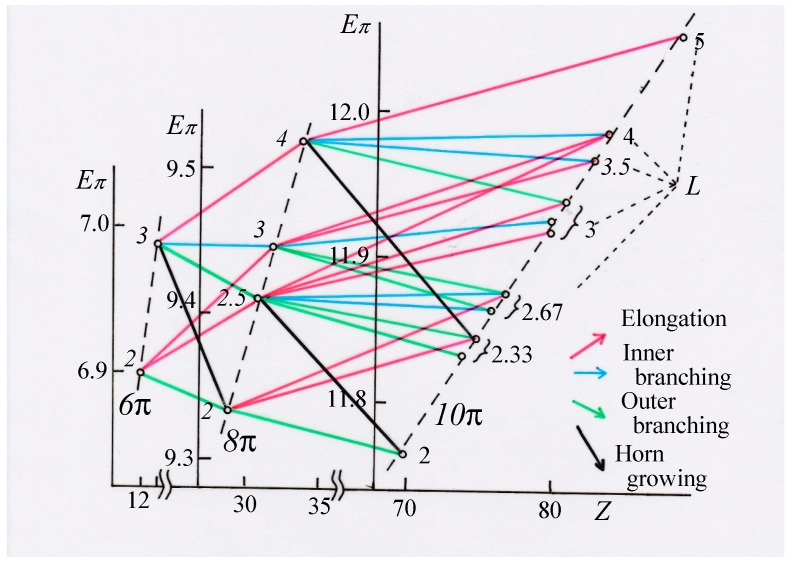
Quantitative diagram demonstrating the systematic growing scheme of the genealogy of C_6_~C_10_ isomers.

It can easily be seen that all these numbers (except for the reverse feature of *T*) lie almost in parallel with each other. Our discussion can be strengthened by drawing a more quantitative diagram such as Figure 7, where although the relative height of the ordinates for the three isomer groups is tentative, their correlation diagrams within each isomer are drawn to the same scale. Here, we can see the four groups of almost parallel arrows representing the growing process by their own colors. For example, see the blue and green lines connecting C_8_ and C_10_, indicating that the *E*_π_ values of different isomers of C_10_ derived by inner branching from C_8_ are found to lie within a small range and distinctively different from the isomers derived by outer branching.

Very large destabilization caused by horn growing (black lines) is prominent in this diagram, while stabilization by elongation (red) and destabilization by outer branching (green) can also clearly be perceived. On the other hand, the change by inner branching (blue) is less prominent, but distinctive from the three other types of growing. Actually, we could draw this type of diagrams showing the systematic genealogy of larger isomers up to C_14_, which are unfortunately not given here because of their entangled look.

Before going into more detailed discussion, we can summarize the global features of the structure–stability relation in the genealogy of conjugated acyclic polyenes as follows:

(i) Relative stability among the isomers derived by elongation, branching, and horn growing can roughly be estimated according to their respective Δ*T* value in the reverse of this order.
(ii) Δ*L* can discriminate between the relative stability of isomers derived by inner and outer branching.
(iii) The lesser stability of outer branching relative to inner branching can be attributed to the short-range conjugation caused by the vinyl group addition, in contrast to the wide-range of the inner branching (elaborated upon later).

In any case, as a rough summary of (i)~(iii), we propose Table 3.

Although this is manifest at the level of elementary chemistry, the present author has never encountered this type of discussion in the literature of chemistry from educational to researchers’ levels. As a mathematical chemist, the author has been struggling—considering the present status of chemical education—to make beginners in the field of chemistry realize that they are in the midst of modern science

### 3.2. Discriminative Power of Z

**Figure 8:**
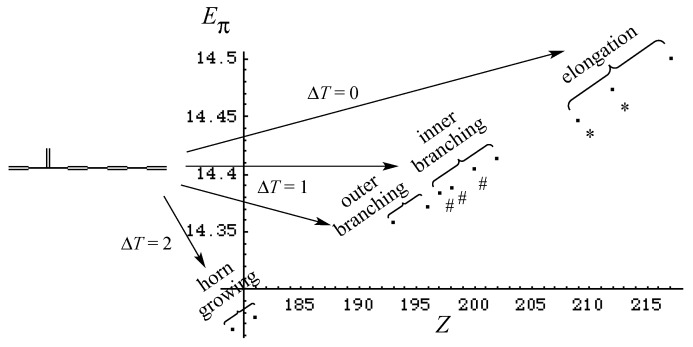
*Z*–*E*_π_ plot of eleven isomers of C_12_ derived from an isomer of C_10_ (5*) showing the four distinctive types of polyene growing. See the later discussion on the marks * and #.

Similar to but more quantitative than Figure 4, we can single out the *Z*–*E*_π_ plot for the C_12_ isomers derived from an isomer of C_10_ (let us call 5* here) as shown in Figure 8.

**Figure 9:**
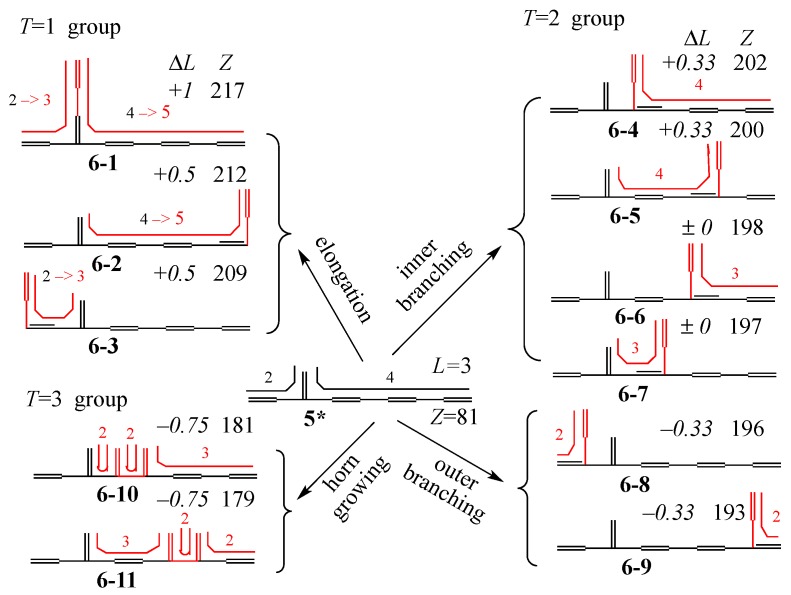
Diagram showing the change of conjugated paths (in red) in each C_12_ isomer caused by the addition of a vinyl group and horn growing to 5*. Δ*L* (in italics) and *Z* for each isomer are also given.

In order to supplement the discussion more quantitatively, we have prepared Figure 9, where for each C_12_ isomer the changed and/or added conjugated path caused by the vinyl addition and horn growing is drawn by the bent line in red, by which Δ*L* can be calculated. The *Z* values of the eleven C_12_ isomers are also given. Notice that as their stability (*E*_π_) shown in Figure 8 is well correlated with *Z*, it is very easy to locate the point for each isomer in Figure 8.

The C_10_ isomer 5* selected for Figure 8 and Figure 9 is situated as the fourth most stable 10π polyene with *L* = 3 in Figure 7. By overlapping eleven diagrams similar to Figure 8 for all the isomers of C_10_, we can obtain the complete genealogy diagram for the relation among all the isomers of C_10_ and C_12_. However, Figure 8 and Figure 9 by themselves reveal very important secrets underlying the whole genealogy together with interesting issues for grading the various theories or indices for discussing this structure–stability problem.

Further, Figure 8 disclosed the limitation of Hess–Schaad recipe [30] for reproducing the *E*_π_ value of conjugated acyclic polyenes. If one calculates the *E*_π_ of the two and three isomers marked with * and # in Figure 8, respectively, the same values can be obtained in each group. This redundancy already occurs for two pairs in the eleven isomers of C_10_, indicating that the Hess–Schaad recipe is only applicable to small polyenes. Actually, for the three kinds of elongated C_12_ isomers in Figure 8, their stability difference is so large that it should not be overlooked.

**Figure 10:**
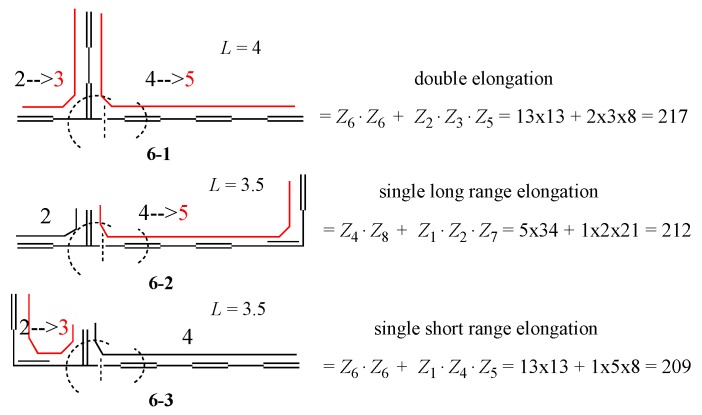
Back-of-envelope calculation of *Z*-indices of **6-1**~**6-3**, where *Z_n_* means the *Z* of path graph S_*n*_, or *n*-th Fibonacci number (See Appendix A). First cut the graph at the dashed straight line, then cut at the dashed round curve to apply the recursion formula. Bent lines are the same as in Figure 9.

If one uses the recursion formula explained in Appendix A, the *Z* values of **6-1**~**6-3** can be obtained by a “back of envelope” calculation as in Figure 10, where bent lines are reproduced from Figure 9 for calculating the *L* values, which cannot discriminate the different stability between **6-2** and **6-3**. However, one can guess that this stability difference might come from the difference between the “long range elongation” and “short range elongation” caused by the vinyl addition. On the other hand, **6-1** obtains the largest stabilization by “double elongation”. Similar results can be obtained by selecting many other polyene isomers for the group with the same *T* value. Thus, we can safely assert that

(iv) *Z*-index can accurately discriminate the stability difference among the same type of growing process, while Δ*L* cannot.
(v) Deep understanding of the structure–stability relation of conjugated acyclic polyenes can be obtained by the complementary discussion with their *T*, *L*, and *Z* values, even without the help of a computer.

### 3.3. Beyond Conjugated Acyclic Polyenes

**Figure 11:**
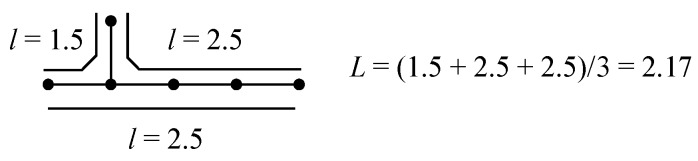
Counting of *L* for a conjugated acyclic polyene radical.

Up to now, we have discussed only the “stable” conjugated acyclic polyenes. Then, consider the relevant radicals, such as 
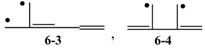
, and 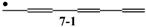, etc.? Actually, the *Z*s of **6-3** and **6-4** are calculated to be 11 and 10, respectively, in parallel with their low *E*π values of 6.159 and 6.000 relative to their singlet isomers, **6-1** and **6-2**. While we can systematically discuss the stability of all these 6π conjugated systems by using their *Z* and *T* values, we are confronted with the difficulty of counting *L* values for radicals. However, try to expand our definition of *L* to conjugated acyclic polyene radicals as in Figure 11, where we take the average *L* of the length of conjugation *l* for all the possible paths in the given radical. This time, the value of *l* composed of *b* CC bonds is tentatively chosen as (*b* + 1)/2, which is consistent with what has already been defined for stable (singlet) conjugated acyclic polyene molecules.

**Figure 12:**
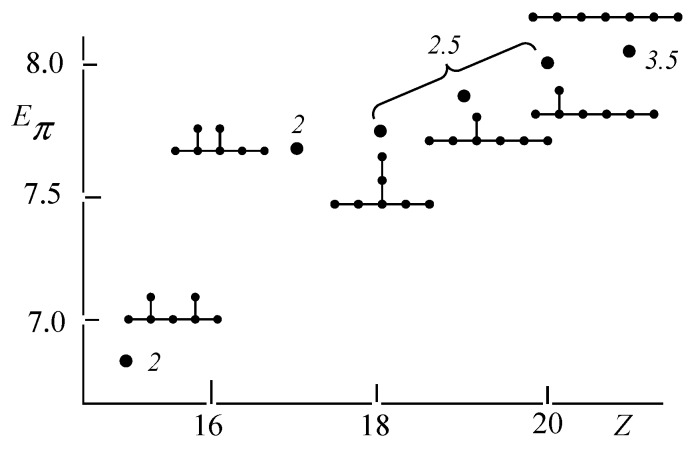
Z–*E*_π_ plot of six isomers of C_7_H_9_ conjugated radicals, among which only one isomer is a quartet radical.

Now, consider the family of C_7_H_9_ conjugated radicals. See Figure 12, where *E*_π_ values of the six isomers are plotted against *Z* and their *L* values are written down in italics. Only one isomer situated as an outlier is in quartet ground state, while all others in doublet ground state are plotted on a straight line.

Since all these heptatrienyl radical family members can be derived from the hexatriene family by methyl addition to either of **6-1**~**6-4**, we can draw diagrams such as those in Figure 6 and Figure 7 in the case of vinyl addition. Although the results are not given here, the global features of the genealogy of this case are very similar and our findings (i)~(v) can be applied.

In this way, the genealogy of conjugated acyclic polyene molecules and radicals is shown to be governed by rather simple rules, which can be explained roughly by naive chemical tools as *T* and *L*, but supported profoundly by the *Z*-index.

Although the present author has already analyzed the concept of aromaticity and anti-aromaticity for conjugated cyclic compounds by using the modified *Z*-index in line with the present analysis [4], the results obtained in the present analysis would be helpful for supplementing and remolding the previous theory. This work is in progress.

**Figure 13:**
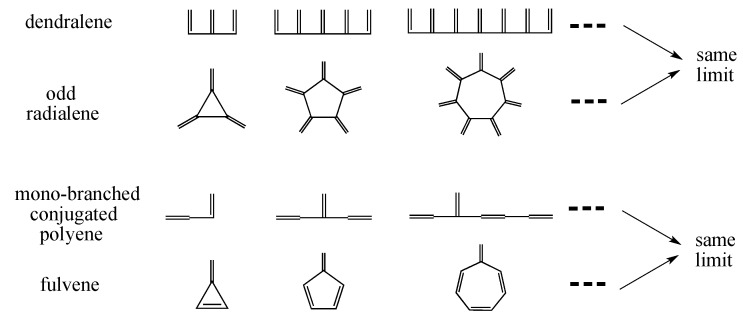
Infinitely large odd radialenes and fulvenes, respectively, converge to the limit of certain cross-conjugated acyclic polyenes.

However, before tackling this big problem, i.e. aromaticity, one needs to extend the definition of conjugated acyclic polyenes and also change the conventional definition of cross-conjugation. See Figure 13, which demonstrates that the following two pairs of conjugated polyenes, namely (a) dendralene and (odd) radialene, and (b) a certain kind of mono-branched conjugated polyene and fulvene, are approaching the same limits, respectively. This means that both of these two pairs of conjugated polyenes should belong to the same family, or “conjugated acyclic polyenes”. If the term “acyclic” is not favorable, one may rephrase it as “conjugated mono-Kekulenoid polyenes” or simply as “conjugated polyenes”. In any event, we should repel an even-membered cycle. Also, keep in mind that only a single odd-membered cycle is allowed, since a couple of disjoint odd-membered cycles contributes a small amount of aromatic or anti-aromatic character to the π-electron system [14].

As already inferred in the above discussion, one may notice that radialenes and fulvenes are automatically joining the club of “cross-conjugated” hydrocarbons. That is, the conventional definition of cross-conjugation indicates such “a compound possessing three unsaturated groups, two of which, although conjugated to a third unsaturated center, are not conjugated to each other” [15]. However, as Hopf already declared [7], let us use the term cross-conjugation in a broader sense than defined above. Namely, if at least one C=C double bond in a molecule is conjugated with more than two conjugated paths, that molecule has a cross-conjugated π-electron system. Then, triafulvene, the smallest fulvene with only four π-electrons, is an important member of the cross-conjugated polyenes and can join the enlarged family of conjugated acyclic polyenes [6].

The present author has already pointed out that by applying the idea of *Z* and *L* to conjugated polyene networks, we can obtain mathematical support for and point out the limitation to the conventional organic electron theory, especially the use of the “curved arrow” originally proposed by organic chemists without any knowledge of quantum mechanics. However, here, we do not expand the scope of the discussion to these issues. The interested readers can refer to the relevant papers [1,2,4,6].

## Figures and Tables

**Table 1:** Number of isomers of conjugated acyclic polyenes C_2*n*_H_2*n*+2_.

*n*	1	2	3	4	5	6	7
No. of isomers	1	1	2	4	11	30	96

**Table 2:** Characteristic quantities of four isomers **4-1**~**4-4** of octatetraenes.

Isomer	Molecular Graph G	*E_π_*	*E*(H&S)	*Z*	P_G_(*x*)	*T*	*L*
**4-1**	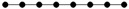	9.518	9.538	34	*x*^8^ − 7*x*^6^ + 15*x*^4^ – 10*x*^2^ +1	0	4
**4-2**	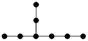	9.446	9.447	32	*x*^8^ − 7*x*^6^ + 14*x*^4^ – 9*x*^2^ +1	1	3
**4-3**	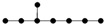	9.409	9.408	31	*x*^8^ − 7*x*^6^ + 14*x*^4^ – 8*x*^2^ +1	1	2.5
**4-4**	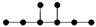	9.332	9.308	29	*x*^8^ − 7*x*^6^ + 13*x*^4^ – 7*x*^2^ +1	2	2

P_G_(*x*) can be obtained by using Equation (5).

**Table 3:** Energy change caused by the four types of growing supported naively by the change of *T* and *L*.

Type of Growing	Energy Change	Δ*T*	*ΔL*
elongation	Stabilization	0	+
inner branching	small change	+1	+, 0, –
outer branching	Destabilization	+1	–
horn growing	big destabilization	+2	– –

**Figure A1:**
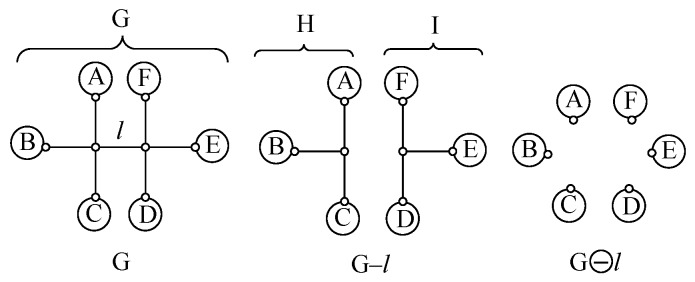
Recursion formula for obtaining *Z* of G by two-step deleting of edge *l*.

**Figure A2:**
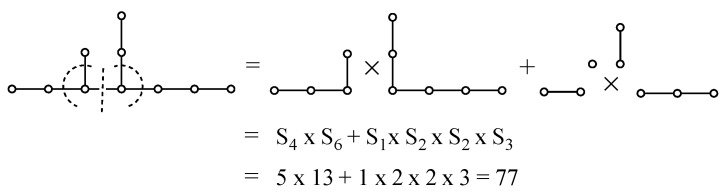
Example of calculating *Z* of a tree graph with ten vertices.

## Appendix A. Simple Recipe for Calculating the *Z*-Index of Branched Conjugated Polyenes

Since the *p*(G,*k*) numbers and *Z*-indices were defined in 1971 [13] and have been discussed so often, here, a simple recipe for the “back-of-envelope calculation” of *Z* for a tree graph will be introduced. First, remember that the *Zs* of path graph S_*N*_, or the carbon atom skeleton of linear polyene or radical with *N* carbon atoms, are nothing but the Fibonacci numbers as given in Table A1. These *Z*s can easily be derived by using the recursion relation,

*Z_N_* = *Z*_*N*–1_ + *Z*_*N*–2_,

together with the initial values of Z_1_ = 1 and Z_2_ = 2. Note also that by rotating Table A1, or the *p*(G,*k*) values for the S_*N*_ family, counter-clockwise by 45 degrees, the famous Pascal’s triangle will appear.

One more important tip is the recursion formula given in Figure A1, where G–*l* is the subgraph of G obtained by deleting an arbitrary edge *l*, and GΘ*l* is the one obtained from G–*l* by further deleting all the edges which were adjacent to *l*. If a graph is divided into several components by either of the above cutting processes, the *Z* value of the resultant multicomponent graph is the product of the *Z*s of all the components as shown in Figure A1. Then, by adding the *Z* values of the two subgraphs, G–*l* and GΘ*l*, we obtain the *Z* value of G.

**Table A1:** *p*(G,*k*) and *Z*-index of path graph S_*N*_.

*N*	G = *S*_N_	*p*(G,*k*)					*Z_N_*
		*k* = 0	1	2	3	4	
1	●	1					1
2		1	1				2
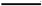
3		1	2				3
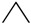
4		1	3	1			5
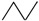
5		1	4	3			8
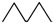
6		1	5	6	1		13
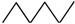
7		1	6	10	4		21
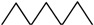
8		1	7	15	10	1	34
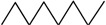

Let us apply this recursion formula to a graph G with ten vertices given in Figure A2. First cut the edge in the middle (the vertical straight dashed line) to obtain G–*l*; then, do the second cut (pair of the curved dashed lines) to obtain GΘ*l*. Take the product sum of all the components according to Figure A1, and we obtain *Z* = 77 for G.
